# Larval Competition Reduces Body Condition in the Female Seed Beetle, *Callosobruchus maculatus*

**DOI:** 10.1673/031.012.3501

**Published:** 2012-03-14

**Authors:** Daynika J. Schade, Steven M. Vamosi

**Affiliations:** Department of Biological Sciences, University of Calgary, 2500 University Drive NW, Calgary Alberta, Canada T2N IN4

**Keywords:** body components, Bruchinae, development, scaled body mass index

## Abstract

Early body condition may be important for adult behavior and fitness, and is impacted by a number of environmental conditions and biotic interactions. Reduced fecundity of adult females exposed to larval competition may be caused by reduced body condition or shifts in relative body composition, yet these mechanisms have not been well researched. Here, body mass, body size, scaled body mass index, and two body components (water content and lean dry mass) of adult *Callosobruchus maculatus* (Fabricius) (Coleoptera: Chrysomelidae: Bruchinae) females exposed to larval competition or reared alone were examined. Experimental females emerged at significantly smaller body mass and body size than control females. Additionally, scaled body mass index and water content, but not lean dry mass, were significantly reduced in experimental females. To our knowledge, these are the first results that demonstrate a potential mechanism for previously documented direct effects of competition on fecundity in female bruchine beetles.

## Introduction

Early body condition has important consequences for fitness (Thornton 2008) and is determined during the crucial time of early life, defined as the period from conception to maturity (Henry and Ulijaszek 1996). From the standpoint of the growing individual, optimal environmental conditions for development include, for example, an abundance of high quality food and space as well as ideal temperatures, humidity, and/or lighting conditions (Prout and McChesney 1985; Vamosi and Lesack 2007; Schirmer et al. 2008). Factors that may result in sub—optimal conditions include exposure to predators (Brodin et al. 2006; Wohlfahrt et al. 2007; Mikolajewski et al. 2008), sexual conflict (Abbott et al. 2010), pollution or feces (Bedhomme et al. 2005), and stress (Shoemaker et al. 2006; Shoemaker and Adamo 2007). Adverse conditions encountered during development can have significant negative impacts on mass at birth or emergence (Metcalfe and Monaghan 2001), metabolic rate (Verhulst et al. 2006), and disease resistance (Reilly and Hajek 2008). Thus, poor early body status may reduce fitness through reduced survival and/or reproductive success (Lindström 1999).

The fitness potential of holometabolous adult insects is often influenced primarily during larval development by resource availability and acquisition ability (Boggs and Freeman 2005). *Callosobruchus maculatus* (Fabricius) (Coleoptera: Chrysomelidae) is a holometabolous insect with larval and pupal stages confined within a bean, which may be shared by several individuals (Ofuya and Agele 1989; Messina and Tinney 1991), followed by a free—living adult form. Because *C. maculatus* do not need to feed or drink as adults to successfully reproduce (e.g., Fox 1993), one can experimentally isolate effects of larval conditions on adult fitness. Furthermore, because there is no parental care, reproductive success is tightly correlated with number and quality of eggs laid. Although the presence of a single competitor may only reduce body mass of *C. maculatus* females (Colegrave 1993), subsequent studies have revealed that females experiencing higher levels of larval competition tend to have a lower body mass upon emergence and lay fewer eggs for their mass than control females (Vamosi 2005; Vamosi and Lesack 2007). The latter results suggest that competition may affect fecundity independent of any effects on mass, but tests of proximate mechanisms are currently lacking.

Prior to proceeding, however, we note that there has been considerable debate of late regarding the way in which body condition is estimated (e.g., Green 2001; Schulte-Hostedde et al. 2005; Peig and Green 2009, 2010). Traditionally, the effects of body size on body mass would first be “controlled for” by obtaining residuals from this regression and then conducting a one—way analysis of variance (ANOVA) on these residuals (i.e., estimates of body condition; Schulte-Hostedde et al. 2005), using competition treatment as the binary predictor variable. However, it has been pointed out (e.g., Green 2001; Peig and Green 2009, 2010) that this method generates biased parameter estimates and, more generally, that the use of residuals as data should be limited to post—hoc diagnosis of model fits (e.g., Garcìa-Berthou 2001; Freckleton 2002). More recently, a new approach based on allometric scaling has been proposed (Peig and Green 2009, 2010; see Materials and Methods for an overview). This method appears especially preferable when attempting to compare body condition of groups that differ in mean size but it has not been previously applied to insects. Here, it was investigated whether larval competition affects body condition of adult females. Females exposed to larval competition were predicted to have lower body condition than those reared alone. It was also tested whether larval competition affects individual body components of adult females. Because adult females exposed to larval competition during development may lay fewer eggs than predicted for their body mass, an associated reduction in relative water content in experimental females was predicted.

## Materials and Methods

### Study organism

The ‘hQ’ strain of *C. maculatus,* which displays a scramble competition strategy in the larval stage (i.e., if several eggs are laid on a single bean, multiple adults may emerge), was used. Stock cultures of beetles were reared on adzuki beans (*Vigna angularis*) and maintained at 28 ^°^C, 50% RH and 24—hour dark conditions in Percival I33LLC8 growth chambers (www.percival-scientific.com).

### Competition treatments

Several hundred adults from the stock culture were allowed to mate and oviposit on beans for 48 hours. Because ovipositing females may be able to recognize low quality beans (Mitchell 1975; Vamosi 2005), each bean was examined after 48 hours and only those with at least three eggs attached were retained for further use. Following previous studies (e.g., Vamosi 2005), two treatments were established (hereafter ‘experimental’ and ‘control’) in which females differed in the intensity of larval competition they experienced during development. Larval competition was manipulated by scraping off unwanted eggs before the larvae hatched and burrowed into beans. Although this method is relatively labor intensive, it avoids confounding effects potentially introduced by having two groups of parental females (i.e., few females on many beans to produce the control group, many females on few beans to produce the experimental treatment). Beans were randomly assigned to the two treatments. Approximately 150 beans had their eggs reduced to one egg per bean, using a scalpel to remove excess eggs. Beans were individually placed in 1.5 mL microcentrifuge tubes, with a small hole punctured in the lid for respiration, for incubation until emergence. This procedure ensured that adults emerging in the control treatment would have experienced no larval competition. For the competition treatment, approximately 250 beans were handled, without the removal of any eggs, and individually placed in similarly prepared 1.5 mL microcentrifuge tubes. More beans were isolated for the competition treatment because pilot studies revealed that the likelihood of a single egg on a bean producing one emerging adult was greater than that of several (i.e., three or more) eggs producing at least three emerging adults.

Beginning 20 days after oviposition, tubes were checked daily for the control group and several times a day for the experimental treatment. Once emergence began, adult females were isolated in microcentrifuge tubes. To ensure that all the females from the experimental treatment were unmated, only females found alone or with other females were considered. All males, as well as females found to have emerged in the same time interval as a male, were recorded and discarded. Once an experimental female was isolated, the level of competition experienced by that female was determined by dissecting the bean to examine it for pupae or adults that had not yet emerged. To ensure larvae from the competition treatment experienced measurable effects of competition (*cf.* Vamosi 2005), only females reared with at least two other individuals that were minimally in the pupal stage when the female emerged were retained. Sample sizes were *N* = 30 for both treatment groups.

### Body components

Procedures for obtaining body component measures followed those of Keller and Passera (1989). Within 24 hours of emergence, females were placed in sealed vials containing a swatch of paper towel wetted with ethyl acetate. The vapor killed the females within minutes and they were subsequently removed with forceps and measured for wet mass (hereafter, body mass) to the nearest 0.01 mg using a Sartorius balance (www.sartorius.com). Immediately upon obtaining body mass of females, three linear body measurements (right elytron length, right elytron width, and pronotum width) were obtained using a Leica microscope (www.leica-microsystems.com). Females were then placed in individual 10 mL glass screw top vials supported within a test tube rack and dried at 70.6 ± 0.4 ^°^C in a Fisher Scientific Isotemp Oven (www.fischersci.com) for 24 hours. To limit the absorption of atmospheric moisture, dry mass of females after water removal was obtained within 15 min of removal from the oven, which was subtracted from body mass to obtain water content. Females were returned to their individual vials and 10 mL of petroleum ether was injected with a syringe into each vial before being returned to the oven for an additional 24 hours. Females were removed from the vials with forceps and placed in clean vials followed by a second 24— hour period of drying. To limit the absorption of atmospheric moisture, lean dry mass was measured for all females within 15 min of removal from the oven. Because no experiments were carried out to ensure that all fat was removed by the procedure (see O'Donnell and Jeanne 1995), results of fat content analyses are not reported.

### Statistical analyses

Although our aim is not to critique the various methods, it was necessary to choose one *a priori,* rather than applying both and presenting the one that produced “significant” results. Because body size is often lower on average in competition females (e.g., Vamosi 2005), this raised the possibility that the slope of the relationship between size and mass would differ between control and experimental groups. Attempting to apply an ordinary least squares approach in such a scenario is problematic whether one assumes a constant slope (because there is evidence that the relationship between size and mass is actually curvilinear; Peig and Green 2010) or allows for two slopes (because the mean of the residuals for each group will necessarily be zero). Following Peig and Green (2009, 2010), three main steps were undertaken to obtain a ‘scaled mass index’ of body condition (hereafter, scaled body mass index) for individuals. First, the body size measurement that was most strongly correlated with body mass was determined. All three linear body measurements and also the first principal component from a principal components analysis (PCA) that included these body measurements (see also Schulte-Hostedde et al. 2005; Colgoni and Vamosi 2006) were included. In agreement with Peig and Green (2009, 2010), one of the single linear body measurements (i.e., right elytron length), and not Principal Component 1 from the PCA, was most strongly correlated with body mass (*r* = 0.76, *t*_58_ = 9.04, *P* < 0.01). Second, In— transformed right elytron length was regressed against In—transformed body mass with standardized major axis regression, to obtain the slope estimate of this relationship (*b*SMA). RMA for Java v. 1.21 (Bohonak and van der Linde 2004) was used for this procedure. Finally, the scaled body mass index (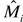 *i* ; mg) for each individual was calculated with:


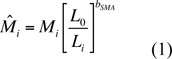


where *M_i_* and *Li* are the body mass and right elytron length of individual *i* respectively, and *L_0_* is the arithmetic mean value for the sample (= 2.01 mm). The effect of the competition treatment on scaled body mass index was analyzed with one—way ANOVA. Correlations between scaled body mass index and scaled body components (water content and lean dry mass) for experimental and control females were calculated. To account for multiple comparisons, a correlation was deemed significant only when *P* < α/4 = 0.0125. Scaled body components were obtained in the same way as described for scaled body mass index, substituting the appropriate body component for body mass in each case. First, multivariate ANOVA (MANOVA) was applied, followed by subsequent univariate ANOVAs for each body component. Analyses of correlations and treatment effects were conducted with R 2.12.1 (R Development Core Team 2010).

## Results

**Figure 1.**
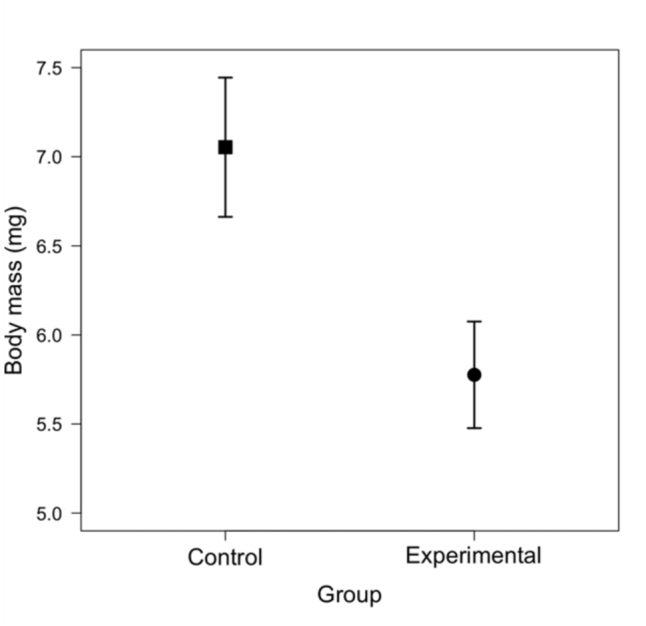
Body mass at emergence (mean ± SE; mg) of control and experimental *Callosobruchus maculatus* females (*N* = 30 for both groups). High quality figures are available online.

**Figure 2.**
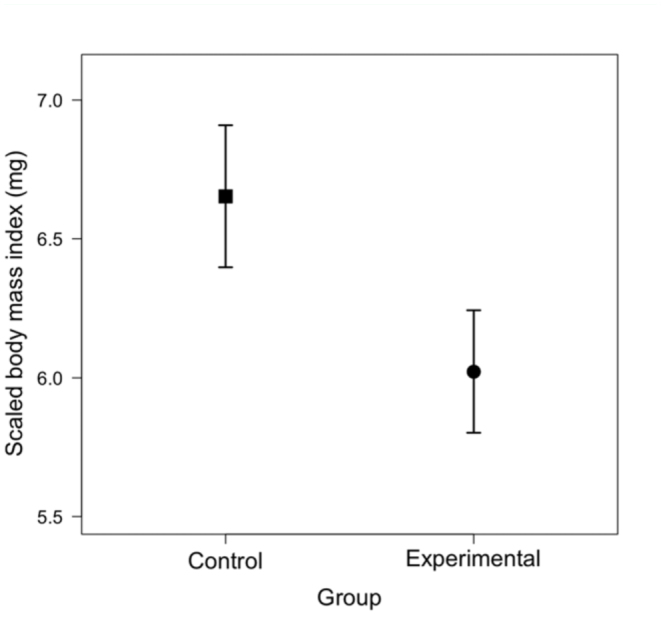
Scaled body mass index (i.e., body condition) at emergence (mean ± SE; mg) of control and experimental *Callosobruchus maculatus* females (*N* = 30 for both groups). Data shown represent female body mass standardized to a length of 2.01 mm (mean right elytron length for the sample). High quality figures are available online.

Experimental females emerged at a significantly lower mean body mass (mean: 5.78 vs. 7.05 mg; *F*_1,58_ = 27.42, *p* < 0.01; Figure 1) and smaller body size (*F*1,58? = 4.90, *p* < 0.05) than control females. The slope of the relationship between In—transformed right elytron length and In—transformed body mass also differed markedly between the two groups (mean ± SE: control females, *b*_SMA_ = 3.24 ± 0.38; experimental females, *b*_SMA_ = 2.42 ± 0.30). One—way ANOVA on scaled body mass index values revealed that experimental females had significantly lower values than control females (*F*_1,58_ = 14.61, *p* < 0.01), with a mean reduction of 9.5% (Figure 2). The findings of reduced mean body mass and scaled body mass index (i.e., body condition) suggest that negative physiological effects of competition were successfully attained by our protocol (see also Vamosi 2005; Vamosi and Lesack 2007).

**Table 1.**
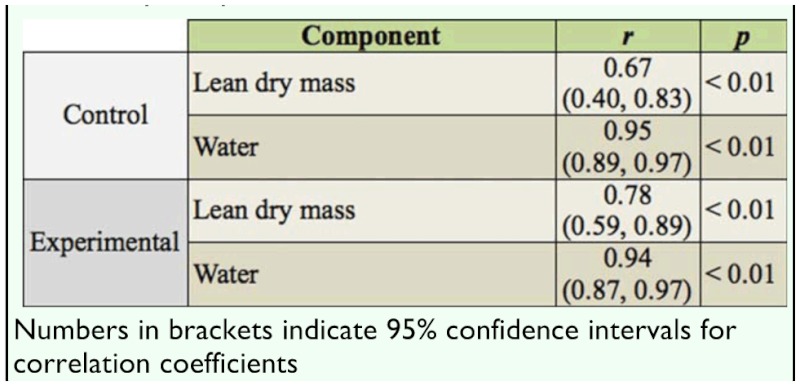
Correlations between scaled body mass index and scaled body components.

All four correlations between body condition and scaled body components were significant, even accounting for multiple comparisons (Table 1). In both groups, the ranking of the correlation between scaled body mass index and body components was the same as observed for five mammal species in Peig and Green (2009; based on original data from Schulte-Hostedde et al. 2005) i.e., water > lean dry mass. Although MANOVA was not significant (F_3,56_ = 1.93, *p* = 0.13), subsequent univariate tests revealed a significant negative effect of competition treatment on scaled water content (*F*1,58* =* 5.05, *p* < 0.05) and a nonsignificant negative effect on lean dry mass (F1,58 = 3.51, *p* = 0.066).

## Discussion

Extending previous studies that demonstrated a reduction in mass—corrected number of eggs laid by females exposed to larval competition (Vamosi 2005; Vamosi and Lesack 2007), scaled body condition and body components of control and experimental females were analyzed. Experimental females were predicted to have reduced body condition and reduced water content compared to control females. Females that were reared with at least two other individuals while developing in medium—sized beans (i.e., experimental females) were significantly lighter and smaller at emergence than those reared alone (i.e., control females), in agreement with previous studies (e.g., Colegrave 1993, Vamosi 2005). Additionally, a significant reduction in body condition was observed, measured as scaled body mass index (Peig and Green 2009, 2010), in experimental females. With regard to body components, there was a significant reduction in water content (mean effect = -6.7%) and a marginal reduction in lean dry mass (-7.3%) in experimental females. To our knowledge, this is the first investigation and documentation of potential mechanisms that may cause previously documented direct negative effects of competition on fecundity in bruchine beetles (Vamosi 2005; Vamosi and Lesack 2007).

Water availability has been shown to affect various aspects of the biology of bruchine beetles. Bruchine beetles are classified as being xerophilic (i.e., able to grow and reproduce without access to free water; Appel et al. 2009), although they will drink free water and lap at sugar—water (e.g., Fox and Moya- Laraño 2009; D Schade and SM Vamosi, pers. obs.). Contrary to expectations, female bruchine beetles do not preferentially lay eggs on high moisture seeds, although the apparent preference for dry seeds may simply be because the latter have reduced chemical defenses (Hudaib et al. 2010). Availability of water has been demonstrated to have significant effects on the mating behavior of adult *C. maculatus* females (Edvardsson 2007; Ursprung et al. 2009; Fox and Moya-Laraño 2009). Females provided with access to free water have been observed to mate less frequently than those deprived of water (Edvardsson 2007; Fox and Moya-Laraño 2009). Water, rather than nutrient content, in the ejaculate has been suggested to modulate remating frequency in adult females (Ursprung et al. 2009). Access to water may be associated with significant positive effects on fecundity and longevity of females, although both effects appear strongest when water is provided in combination with sugar (Ursprung et al. 2009; Fox and Moya-Laraño 2009). Together, these observations suggest that the reduction in water content of experimental females documented in the present study may translate into biologically relevant consequences for their mating behavior and fecundity.

Evaluating the reception of the scaled mass index method for estimating body condition is currently difficult, given the lack of studies that have cited Peig and Green (2009, 2010) thus far. However, three observations suggest that it may be a robust methodology for similar studies in future. First, the slopes of the relationship between In—transformed right elytron length and In—transformed body mass for *C. maculatus* females corresponded well to values reported in Table 2 of Peig and Green (2009) for seven vertebrate species (median: 2.9, range = 1.4 to 3.6). Second, correlations between body condition and scaled body components (Table 1) were similar in magnitude to the mean values (lean dry mass: 0.84; water: 0.91) computed for the five mammal species reported in Table 3 of Peig and Green (2009). Finally, and most significantly, this methodology allowed for the comparison of body condition of two groups (experimental vs. control females) that differed in the slope of the relationship between size and mass. It is likely that the method defended by Schulte-Hostedde et al. (2005) will continue to be appropriate in many instances, but we suggest researchers consider applying Peig and Green's (2009, 2010) scaled body mass index for estimating body condition whenever there is an *a priori* reason to suspect that the groups being compared will differ in mean body mass and/or body size. Changes in either or both traits are certainly commonly observed in response to competition (Colegrave 1993; Boggs and Freeman 2005; Vamosi 2005; Harvey et al. 2009), but may also be triggered by variation in several other factors, including temperature (Marti and Carpenter 2008) and resource type (Ueno 2003).

Because *Callosobruchus* is increasingly being used as a model organism in several areas of ecology and evolution (e.g., Fox 1993; Crudgington and Siva-Jothy 2000; Arnqvist and Tuda 2010), future investigations should explicitly examine the consequences for different environmental conditions encountered during development on adult behavior and fitness. The present study could be extended in several ways, from comparative and life history perspectives. For the former, because only a single scramble strain was considered, it may be informative to investigate whether similar patterns hold for multiple contest and competition strains. For the latter, body condition could be non—invasively measured (i.e., by measuring only body length and body mass of females upon emergence), which could be included as a covariate in subsequent analyses of mating frequency, longevity, and mass—corrected fecundity of competition vs. control females. Finally, most studies have considered these phenomena in females, whereas the effects on males have been relatively understudied.

In conclusion, exposure to larval competition during development resulted in adult *C. maculatus* females with significantly lower body mass, body size, scaled body mass index (i.e., body condition), and water content than control females. These results are the first to provide a potential mechanism for reduced mass—corrected fecundity in females exposed to competition during larval development, and corroborate previous demonstrations of a potential positive effect of access to free water on longevity and fecundity in bruchine beetles.

## **Abbreviations**

ANOVA: analysis of variance;
MANOVA: multivariate analysis of variance;
PCA: principal components analysis
